# Ascorbate Suppresses VEGF Expression in Retinal Pigment Epithelial Cells

**DOI:** 10.1167/iovs.18-24101

**Published:** 2018-07

**Authors:** David W. Sant, Vladimir Camarena, Sushmita Mustafi, Yiwen Li, Zachary Wilkes, Derek Van Booven, Rong Wen, Gaofeng Wang

**Affiliations:** 1 John P. Hussman Institute for Human Genomics, Dr. John T. Macdonald Foundation Department of Human Genetics, University of Miami Miller School of Medicine, Miami, Florida, United States; 2 Bascom Palmer Eye Institute, University of Miami Miller School of Medicine, Miami, Florida, United States; 3 Dr. Nasser Ibrahim Al-Rashid Orbital Vision Research Center, University of Miami Miller School of Medicine, Miami, Florida, United States

**Keywords:** VEGF, age-related macular degeneration, ascorbate, retinal pigment epithelium, 5-hydroxymethylcytosine

## Abstract

**Purpose:**

To investigate the impact of ascorbate, via DNA hydroxymethylation, on VEGF expression in retinal pigment epithelial (RPE) cells.

**Methods:**

Dot-blot and hydroxymethylated DNA immunoprecipitation sequencing were applied to evaluate the impact of ascorbate on DNA hydroxymethylation in ARPE-19 cells. RNA sequencing (RNA-seq) was carried out to analyze the transcriptome. Quantitative RT-PCR and ELISA were conducted to examine the transcription and secretion of VEGF from cultured cells. Primary human fetal RPE cells and RPE-J cells were used to verify the effect of ascorbate on VEGF expression. ELISA was used to measure VEGF in the vitreous humor of *Gulo*^−/−^ mice, which, like humans, cannot synthesize ascorbate de novo.

**Results:**

Treatment with ascorbate (50 μM) promoted 5-hydroxymethycytosine (5hmC) generation and changed the genome-wide profiles of 5hmC in ARPE-19 cells. Ascorbate also caused a dramatic shift in the transcriptome—3186 differential transcripts, of which 69.3% are correlated with altered 5hmC in promoters or gene bodies. One of the most downregulated genes was *VEGFA*, which encodes the VEGF protein. The suppression of VEGF by ascorbate is independent of hypoxia-inducible factor 1-alpha (HIF-1α) but correlates with increased 5hmC in the gene body. The decreased transcription and secretion of VEGF by ascorbate were verified in primary human fetal RPE cells. Furthermore, adding ascorbate in the diet for *Gulo*^−/−^ mice resulted in decreased levels of VEGF in the RPE/choroid and vitreous humor.

**Conclusions:**

Ascorbate inhibits VEGF expression in RPE cells likely via DNA hydroxymethylation. Thus, ascorbate could be implicated in the prevention or treatment of diseases such as age-related macular degeneration (AMD).

Vascular endothelial growth factor (VEGF) signaling plays a critical role in pathological angiogenesis in the eye. By eliminating the availability of VEGF, intraocular anti-VEGF therapies are effective for wet age-related macular degeneration (AMD), which is characterized by the presence of choroidal neovascularization.^1^ Using either antibodies (such as Lucentis, Avastin) or soluble decoy receptors (such as Eylea), current anti-VEGF therapies target the interaction between VEGF and its receptors, but not endogenous VEGF expression.

The expression of genes such as *VEGFA*, which encodes the VEGF protein, can be modulated epigenetically by active DNA demethylation. Ten-eleven translocation (TET) cytosine dioxygenases initiate active DNA demethylation by converting 5-methylcytosine (5mC) to 5-hydroxymethylcytosine (5hmC).^2^ In addition to being a cytosine demethylation intermediate, 5hmC is also recognized as a unique epigenetic mark that regulates transcription.^3,4^ TETs belong to the iron and 2-oxoglutarate (2OG, also known as α-ketoglutarate)-dependent dioxygenase family. These enzymes utilize Fe^2+^ as a cofactor and 2OG as a cosubstrate. We and others found that ascorbate (ascorbate anion, the dominant form of vitamin C/L-ascorbic acid under physiological conditions) promotes TET-mediated conversion of 5mC to 5hmC.^5–9^ This finding highlights a new function of ascorbate in modulating the epigenetic control of the genome.^10^

In the absence of ascorbate, the initial hydroxylation catalyzed by TETs may proceed to a maximal level. Along with the hydroxylation of 5mC, the cofactor Fe^2+^ is also oxidized to Fe^3+^ and Fe^4+^. However, these oxidized iron species are catalytically inactive and the absence of Fe^2+^ leads to the inactivation of TETs. When available, ascorbate reduces the oxidized iron species back to catalytically active Fe^2+^. Thus, ascorbate acts as a reducing agent to replenish the active Fe^2+^ and maintain the full enzymatic capacities of TETs.^10^

Oxidative stress, characterized by elevated levels of reactive oxygen species (ROS), is involved in the pathogenesis of AMD.^11^ AMD-associated oxidative stress converts ascorbate to its oxidized form of dehydroascorbic acid (DHA), and thus depletes ascorbate in the retina.^11^ Unlike ascorbate, DHA cannot reduce Fe^3+^ to Fe^2+^ and is therefore not functional as a cofactor for the TET enzymes. Thus, oxidative stress–associated ascorbate deficiency interrupts TET-mediated DNA demethylation, which could change the transcription of certain genes and lead to phenotypic alterations. Studies have shown the benefits of antioxidant cocktails, which include ascorbate, in decreasing the risk of AMD.^12–15^ However, it remains elusive whether and how ascorbate decreases the risk of AMD.

In the present work, we investigated the impact of ascorbate on DNA hydroxymethylation and the transcriptome of RPE cells, especially the expression of genes relevant to AMD. We found that ascorbate supplementation significantly suppressed the expression of VEGF in cultured RPE cells including human primary fetal RPE cells and in rodent eyes. Our results highlight the importance of ascorbate as a potential factor in prevention and treatment for VEGF-mediated diseases such as AMD.

## Methods

### Cell Culture

Human retinal pigment epithelial ARPE-19 and rodent RPE-J cells were purchased from American Type Culture Collection (ATCC, Manassas, VA, USA). ARPE-19 cells and RPE-J cells were maintained in DMEM-F12 medium (Life Technologies, Carlsbad, CA, USA) or DMEM medium (ATCC), respectively, which do not contain any ascorbate in their formulation. After seeding approximately 3 × 10^5^ cells in six-well plates or 10^6^ cells in 10-cm plates for 24 hours, they were treated with sodium ascorbate or L-ascorbic acid (Sigma-Aldrich Corp., St. Louis, MO, USA) at different concentrations for varying durations. Primary human fetal retinal pigment epithelial cells (hfRPE) were a generous donation from the Sheldon Miller lab at the National Eye Institute (NEI). Upon receipt, cells were passaged one time and kept in MEM medium (Cat. #5650, Sigma-Aldrich Corp.) with added N1 medium supplement, taurine, hydrocortisone, and triiodo-thyronin (Sigma-Aldrich Corp.) as well as GlutaMAX supplement (Thermo Scientific, Waltham, MA, USA).^16^ Cells were left in culture for 6 weeks and checked for high levels of pigmentation to ensure native RPE characteristics were present before treatment with sodium ascorbate for up to 7 days. Each treatment group consisted of at least three wells or three plates for every experiment. In all experiments cell culture media were changed daily with or without ascorbate to maintain a constant concentration of ascorbate. Each experiment was repeated at least three times.

### 5hmc Dot-Blot Assay

Genomic DNA was extracted from cultured ARPE-19 cells using QIAamp DNA mini kits (Qiagen, Hilden, Germany) according to the manufacturer's instructions. A Qubit Fluorometer (Life Technologies) was used to quantify the concentration of DNA. The dot-blot was performed following a previously published protocol using two spots per sample, one with 0.4 μg and the other with 0.8 μg, to allow for visualization of contrast between both low and high samples.^5^ To ensure equal loading, the membrane was stained with methylene blue post immunoblotting. The densities of the dots on membrane were captured by ImageJ (National Institutes of Health, Bethesda, MD, USA). Statistical significance of differences in 5hmC content between treatments and control was assessed by Student's *t*-test, at α = 0.05.

### hMeDIP-seq

ARPE-19 cells were cultured in confluent monolayer and treated with or without sodium ascorbate (50 μM) for 10 days. Genomic DNA was extracted from ARPE-19 cells using QIAamp DNA mini kits from Qiagen according to the manufacturer's instructions. A Qubit Fluorometer from Life Technologies was used to quantify the concentration of DNA. A Bioanalyzer 2000 (Agilent, Santa Clara, CA, USA) was used to measure the quality of DNA. DNA was submitted for hydroxymethylated DNA immunoprecipitation sequencing (hMeDIP-seq) to the Epigenomics Core at the University of Michigan using previously published protocols.^17^ Briefly, DNA was sonicated to approximately 100 base pairs (bp), ligated with Illumina (San Diego, CA, USA) adaptors, and immunoprecipitated using anti-5hmC antibody (#39791; Active Motif, Carlsbad, CA, USA). DNA of both immunoprecipitated and un-immunoprecipitated (input control) samples was subsequently sequenced on a HiSeq4000 sequencing system (50 bp single-end reads, three samples per lane; Illumina). Analysis of hMeDIP-seq was carried out using the previously reported pipeline.^18^ Briefly, after quality control, reads were aligned using the Burrows-Wheeler Aligner and peaks were called using MACS2, then filtered using the irreproducible discovery rate (IDR) pipeline.^19–21^ Reads within peak regions were quantified using HTSeq-count (available in the public domain, https://htseq.readthedocs.io/en/release_0.10.0/). Statistical significance was determined using edgeR, and only peaks below an adjusted *P* value (false discovery rate, FDR) of 0.05 with a minimum of a 2× fold change were considered differential to minimize false positives.^22^ By this method, 56,487 peaks were upregulated and 7591 peaks were downregulated after treatment with ascorbate while 68,878 peaks remained unchanged. Heatmaps of read density for peaks in each sample were generated using version 2 of DeepTools.^23^

### RNA-seq Analysis

ARPE-19 cells were cultured into a confluent monolayer and treated with or without sodium ascorbate (50 μM) for 7 days. Total RNA was extracted from the cells using the RNeasy Mini Kit (Qiagen). A Bioanalyzer 2000 was used to measure the quality of RNA. All samples' RNA integrity numbers (RIN) were above 9. Whole-transcriptome sequencing (RNA-seq) was carried out at the Sequencing Core of John P. Hussman Institute of Human Genomics at the University of Miami using the Epicentre Ribo-Zero Human/Mouse/Rat kit (Epicentre, Madison, WI, USA). Briefly, after ribosomal RNA (rRNA) was depleted, sequencing libraries were constructed following the standard Illumina protocols and were subsequently processed by a Hiseq2000 sequencing system (125 bp paired-end reads, four samples per lane; Illumina). Data were analyzed using a previously published pipeline.^18^ Briefly, after quality control, reads were aligned to the human transcriptome (GRCh38; Ensembl.org; in the public domain) and quantified using the STAR aligner.^24^ Statistical significances were determined using two different differential expression calculators: edgeR and DESeq2.^22,25^ To reduce false positives, only genes that achieved an adjusted *P* value below 0.05 across both methods were considered “differential.” Read density at the *VEGFA* genomic region was visualized using the UCSC genome browser.^26^

### Quantitative Real-Time RT-PCR

RNA was extracted from cultured cells using RNeasy kits (Qiagen). A nanodrop 8000 photospectrometer was used to measure the yield of RNA extraction (Thermo Scientific). The qScript Flex cDNA kit (Quanta Biosciences, Beverly, MA, USA) was used for reverse transcription (RT) according to the manufacturer's instructions. Quantitative real-time RT-PCR (qRT-PCR) was performed in triplicate on an ABI 7900 (Life Technologies) using the PerfeCTA SYBR Green FastMix ROX (Quanta Biosciences) with 10-μL reactions. Primers were designed to span introns (Supplementary Table S1). The transcript amplification results were analyzed with the ABI 7900 HT software (SDS) (Thermo Scientific), and all values were normalized to the levels of the ACTB using the 2^−(ΔΔCt)^ method. Statistical significance of differences in expression levels was assessed by Student's *t*-test, at α = 0.05.

### ELISA Assay of Conditioned Media

Cell culture media were saved from the final 24 hours of treatment of ARPE-19 or hfRPE cells with sodium ascorbate for varying durations and at varying concentrations. VEGF levels in the cell culture conditioned medium were measured using Human VEGF DuoSet ELISA kits (R&D Systems, Minneapolis, MN, USA) with antibodies mainly specific for VEGF-165 and VEGF-121, the two major isoforms of proangiogenic VEGF, according to the manufacturer's instructions. Differences were assessed by either Student's *t*-test or 1-way ANOVA with a significance level of α = 0.05.

### Immunoblot

ARPE-19 cells were grown in DMEM:F12 medium (Thermo Scientific) with 10% fetal bovine serum (FBS) and pretreated for 7 days with or without ascorbate (50 μM). For the last 6 hours before collection, some wells of both ascorbate-treated and untreated cells were treated with 100 μM cobalt chloride (CoCl_2_) to induce a hypoxia-like state as has been performed previously.^27^ Cell lysates were collected in radioimmunoprecipitation assay buffer (RIPA) buffer (Thermo Scientific) with protease inhibitor cocktail (Sigma-Aldrich Corp.), 1% SDS, and 0.5 mM dithiothreitol (DTT). The protein concentration was determined by Pierce BCA protein assay kit (Thermo Scientific). Cell lysates were resolved by SDS-PAGE, transferred to polyvinylidene fluoride (PVDF) membranes (Bio-Rad Laboratories, Hercules, CA, USA), and immunoblotted with anti-hypoxia-inducible factor 1-alpha (anti-HIF-1α) antibody and anti-β-actin antibody (#3716S and #4970S; Cell Signaling Technology, Danvers, MA, USA). Proteins were visualized by using chemiluminescence using SuperSignal West Femto ECL (Thermo Scientific). Specific band densities were quantified by using ImageJ and analyzed by Student's *t*-test, at α = 0.05.

### Animal Study

All mouse experiments were performed in accordance with the guidelines for the care and use of laboratory animals published by the National Institutes of Health and in accordance with the ARVO Statement for the Use of Animals in Ophthalmic and Vision Research. The use of the animals and the research protocol were approved by the IACUC (Institutional Animal Care and Use Committee) at the University of Miami. *Gulo*^−/−^ mice were obtained from the Mutant Mouse Regional Resource Center (University of California, Davis, CA, USA).^28^ *Gulo*^−/−^ mice (1 month old) were maintained for 3 months with either high ascorbate (330 mg/L of L-ascorbic acid, *n* = 4) or low ascorbate (16.5 mg/L, *n* = 5) in drinking water. After euthanasia of the mice, the mouse heads in their entirety were fixed in 4% paraformaldehyde and stored in phosphate-buffered saline at 4°C until further processing. The eyes were then removed, and the RPE/choroid layer was dissected. Total RNA was extracted from pooled RPE/choroids from a single mouse using the RecoverAll Total Nucleic Acid Isolation kit (Thermo Scientific). The qScript Flex cDNA kit (Quanta Biosciences) was used for RT according to the manufacturer's instructions. Quantitative real-time RT-PCR was performed in triplicate on a QuantStudio 12K Flex (Thermo Scientific) using PowerUp SYBR Green Master Mix (Thermo Scientific) with 10-μL reactions. Primers were designed to span introns (Supplementary Table S1). The transcript amplification results were analyzed with the QuantStudio software (Thermo Scientific), and all values were normalized to the levels of the *Actb* using the 2^−(ΔΔCt)^ method.

In a separate experiment, *Gulo*^−/−^ mice (3 months old) were maintained for 4 months with or without ascorbate (330 mg/L of L-ascorbic acid) in drinking water (*n* = 3 per group). After euthanasia of the mice, the vitreous humor from one eye was extracted and stored at −80°C until further processing. VEGF protein levels were measured in the vitreous humor samples using the Quantikine ELISA Mouse VEGF (R&D Systems) according to manufacturer's instructions. Briefly, 2 μL vitreous humor (from a single eye) was diluted in 8 μL RIPA buffer containing protease inhibitors (Millipore Sigma, Burlington, MA, USA). This 10 μL of this diluted vitreous humor was added to 40 μL sample diluent and loaded to the plate, along with an additional 50 μL assay diluent, followed by a 2-hour incubation. Following a wash step, a conjugate solution was added to the wells for another 2 hours, followed by another wash step. A substrate was added to the wells for 30 minutes, and the reaction was halted with a stop solution. The absorbance was read at 450 nm with a correction wavelength of 540 nm. Statistical significance of differences in expression levels was assessed by Student's *t*-test, at α = 0.05.

## Results

### Ascorbate Causes a Dramatic Shift in the Hydroxymethylome

**Figure 1:**
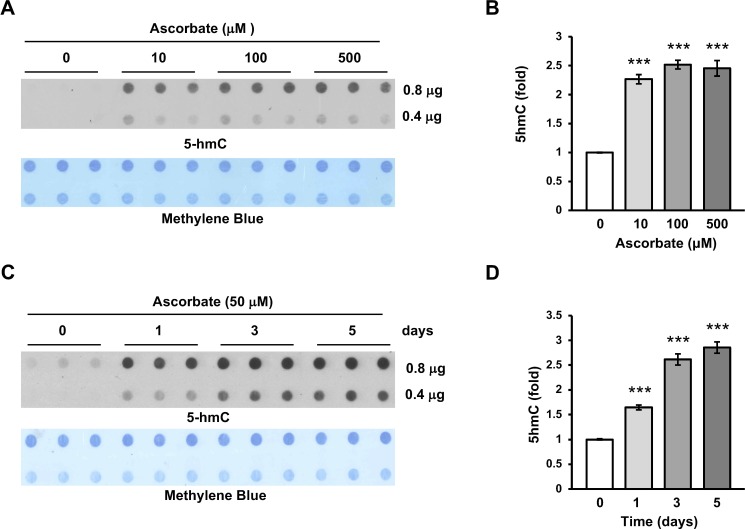
Induction of 5hmC in ARPE-19 cells by ascorbate treatment. (A, B) Dot-blot and semiquantitative analysis of the dot-blot show that ascorbate (0, 10, 100 μM) dose-dependently induced 5hmC in ARPE-19 cells. Ascorbate at 500 μM did not cause further increase in 5hmC, possibly due to its toxicity to the cells at this concentration. Methylene blue staining was used as a loading control. (C, D) Dot-blot and semiquantitative analysis of the dot-blot show that ascorbate (50 μM) time-dependently (0, 1, 3, and 5 days) induced 5hmC in ARPE-19 cells (***P < 0.001).

ARPE-19 cells, derived from human RPE, have been widely used as a RPE cell model and are typically cultured in DMEM:F12 medium, which does not contain any ascorbate in its formulation. Previously, we and others have shown that ascorbate promotes TET-mediated DNA hydroxymethylation.^5–9^ In this study, we first assessed the impact of ascorbate on DNA hydroxymethylation in ARPE-19 cells. We found that 5hmC was barely detectable in DNA from a confluent ARPE-19 monolayer. Addition of ascorbate at different concentrations (10, 100, 500 μM) for 3 days induced a ∼2.5-fold increase in the global content of 5hmC (Figs. 1A, 1B). The average concentration of ascorbate in the plasma of healthy humans is ∼50 μM and can reach up to ∼150 μM.^29^ Ascorbate at a high pharmacological concentration (500 μM) did not exert additional benefits in 5hmC generation. Furthermore, ascorbate increased 5hmC in a time-dependent fashion. The 5hmC content increased to approximately 1.5-fold after 1 day, ∼2.5-fold after 3 days, and ∼3-fold after 5 days (Figs. 1C, 1D). Together, these results indicate that ascorbate profoundly altered the global content of 5hmC in ARPE-19 cells.

**Figure 2:**
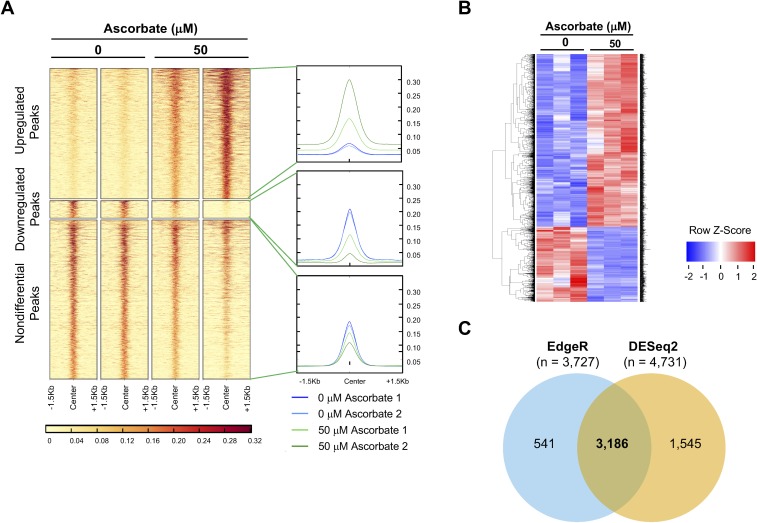
hMeDIP-seq analysis and RNA-seq analysis of ARPE-19 cells treated with or without ascorbate (50 μM). (A) Heatmap of the 5hmC-enriched regions investigated for differential analysis with edgeR. Only regions below an FDR of 0.05 with a minimum of a 2× fold change (below 0.5× or above 2× compared to control) were considered differential. Darker color represents greater coverage in read counts per million (RCPM). Overall coverage plots (right side of heatmap) show the average coverage in RCPM across all peaks. Blue lines represent samples treated with 0 μM ascorbate, and green lines represent samples treated with 50 μM ascorbate. (B) The heatmap shows differentially expressed genes in ARPE-19 cells treated with or without ascorbate (50 μM). Colors represent Z-scores, where downregulated transcripts are represented as blue and upregulated transcripts are represented as red. Only genes determined to be significant by both statistical algorithms (edgeR and DESeq2) are included in the heatmap. (C) Venn diagram showing the number of genes called as statistically significant by the two different statistical algorithms, DESeq2 and edgeR.

The global increase in 5hmC is not expected to be evenly distributed, but rather thought to be indicative of local changes in many small regions of the genome. To investigate the locations of change in the hydroxymethylome, we performed hydroxymethylated DNA immunoprecipitation sequencing (hMeDIP-seq) on DNA from ARPE-19 cells treated with or without ascorbate (50 μM) (Figs. 2A, 2B). Overall, 132,956 regions of the genome were found enriched for 5hmC. Peaks were considered differential if they were given an FDR below 0.05 by edgeR and showed at least a 2-fold increase or decrease.^22^ Using this method, 56,487 peaks were found to have significantly higher enrichment of 5hmC, and only 7591 peaks were found to have significantly lower enrichment of 5hmC in samples treated with ascorbate. The remaining 68,878 regions were found to have similar enrichment across groups (Fig. 2A). These results confirmed that ascorbate treatment indeed increases 5hmC in the genome of ARPE-19 cells, consistent with the results of the dot-blot assays.

### Ascorbate Treatment Alters the Transcriptome of ARPE-19 Cells

An increase in the global content of 5hmC indicates a shift in the dynamic process of DNA methylation-demethylation, which consequently affects transcription. Further, the relatively stable 5hmC itself also can regulate gene transcription as a unique epigenetic mark.^3,4^ Thus, ascorbate-induced 5hmC changes could alter the transcriptional pattern of ARPE-19 cells. To investigate the impact of ascorbate on the transcriptome, we employed transcriptome sequencing, also known as RNA-seq, on ARPE-19 cells treated with or without ascorbate (50 μM). A significant shift in the transcriptome was observed in correlation with the ascorbate-induced increase in 5hmC, as shown in the heatmap of differentially expressed genes (Fig. 2B). A total of 4731 genes were found differentially expressed by DESeq2, and 3727 genes were found differentially expressed by edgeR. A total of 3186 genes were considered significant by both differential expression calculators (Fig. 2C). Of the 3186 genes, the expression of 964 genes was reduced and the expression of 2222 genes increased, which is consistent with the known bidirectional effects of 5hmC on transcription.^30^ Of the 3186 genes, 2208 (69.3%) of them contained a differential 5hmC peak within the gene body or promoter region.

### Ascorbate Treatment Suppresses the Expression of VEGF

**Figure 3:**
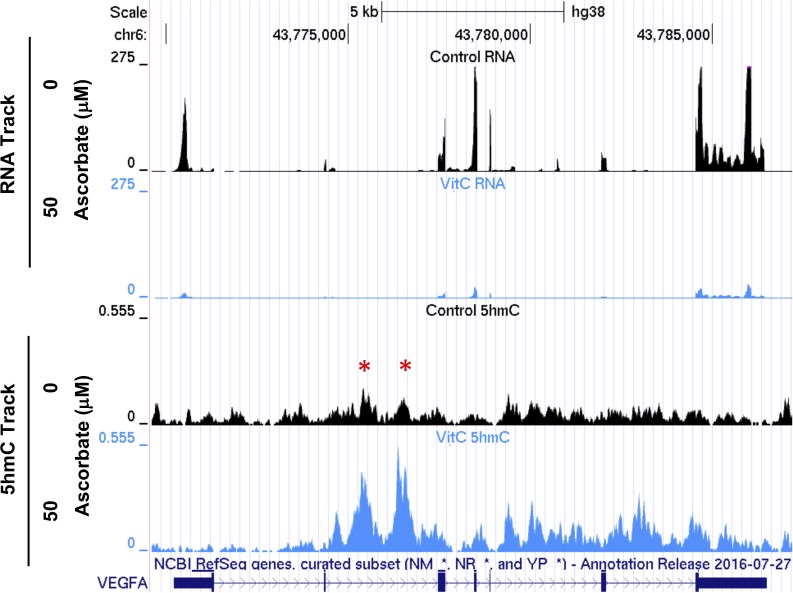
5hmC increases in the gene body of VEGFA after ascorbate treatment. UCSC tracks show the coverage of the RNA-seq and hMeDIP-seq (5hmC) over the VEGFA gene region. The two red stars indicate the 5hmC peaks that increase with 50 μM ascorbate treatment.

Surprisingly, *VEGFA*, which encodes the VEGF protein, was one of the top 10 genes whose transcription was significantly altered by ascorbate treatment. RNA-seq data suggested that ascorbate treatment decreased the expression of *VEGFA* to ∼11% of control level (*P* = 2.9 × 10^−71^). As previously discussed, ascorbate induced a large shift in the hydroxymethylome, and alterations of 5hmC in the *VEGFA* genomic region could lead to changes in *VEGFA* transcription. Upon investigation of the hMeDIP-seq data we found that three peaks of 5hmC enrichment were called in the *VEGFA* genomic region using the MACS2-IDR pipeline. Two peaks in intron 2 increased with 50 μM ascorbate treatment (FDR = 0.0177, 0.0041, respectively). A large peak was detected from intron 5 through the 3′UTR (untranslated region) but was not called as differential (Fig. 3; Table).

**Table:**
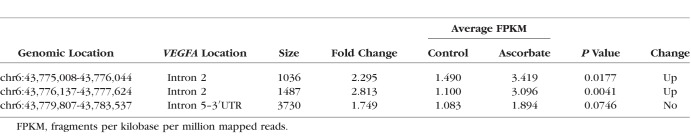
hMeDIP-seq Peaks in the VEGFA Region

**Figure 4:**
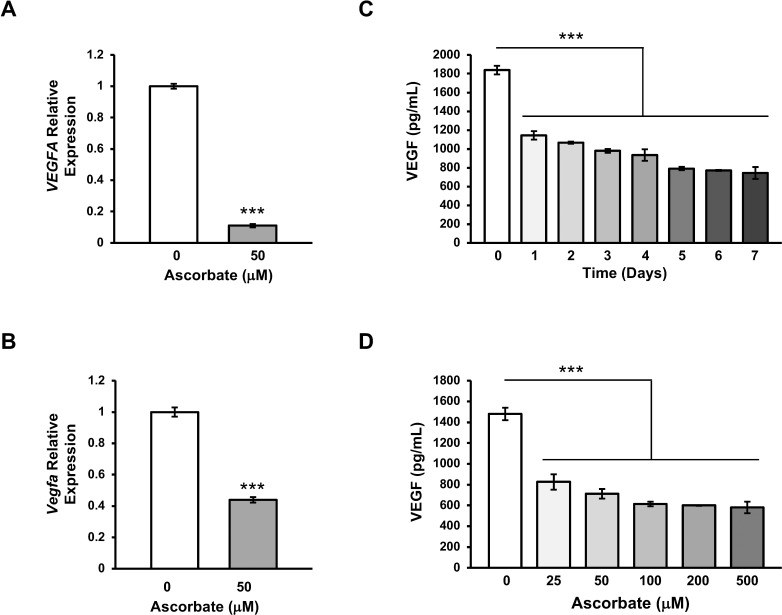
qRT-PCR and ELISA verification of the suppression of VEGFA by ascorbate. (A) The inhibition of ascorbate on VEGFA transcription was validated in a second batch of ARPE-19 cells treated with ascorbate (50 μM). (B) The transcription of Vegfa (homologue of VEGFA) in RPE-J cells was inhibited by ascorbate (50 μM) treatment. (C) ELISA results show that ascorbate time-dependently decreases the levels of VEGF protein in the conditioned culture medium. (D) ELISA testing shows that ascorbate dose-dependently decreases the levels of VEGF in the conditioned culture medium (***P < 0.001).

The initial RNA-seq finding was then confirmed by qRT-PCR in multiple experiments, which indicated a decrease in *VEGFA* mRNA to 13% of the control by ascorbate treatment (Fig. 4A). It is known that *VEGFA* can produce two families of transcripts—the “a-isoform” with proangiogenic function and “b-isoform” with anti-angiogenic capacity. The expression levels of the two isoforms were assessed by qRT-PCR using isoform-specific primers. Results showed that the a-isoform was ∼25-fold more abundant than the b-isoform in cells cultured without ascorbate. After treatment with ascorbate, transcripts of both isoforms were significantly reduced (Supplementary Fig. S1).

RPE-J cells, a rodent RPE cell line, were then used to validate this suppression of *VEGFA* transcription by ascorbate in another cell line. Although ascorbate is not a vitamin for rodents, RPE cells in vitro rely on ascorbate that is endogenously synthesized in the liver. Quantitative RT-PCR analysis of *Vegfa* (homologous to human *VEGFA* gene) expression showed that the level of *Vegfa* transcripts was decreased to ∼44% after treatment with ascorbate (50 μM) for 6 days (Fig. 4B).

To examine whether the reduced VEGF transcription leads to a decrease in VEGF protein secreted from the cells, we employed ELISA to test conditioned medium of ARPE-19 cells. ELISA assays indicated that ascorbate treatment reduced the secretion of VEGF in a time-dependent (Fig. 4C) as well as dose-dependent manner (Fig. 4D). Overall, these results suggest that ascorbate treatment inhibits the expression of VEGF, resulting in lower VEGF protein synthesis and secretion in RPE cells.

### Suppression of VEGF Is Not Correlated With Antioxidant Effects or HIF-1α

**Figure 5:**
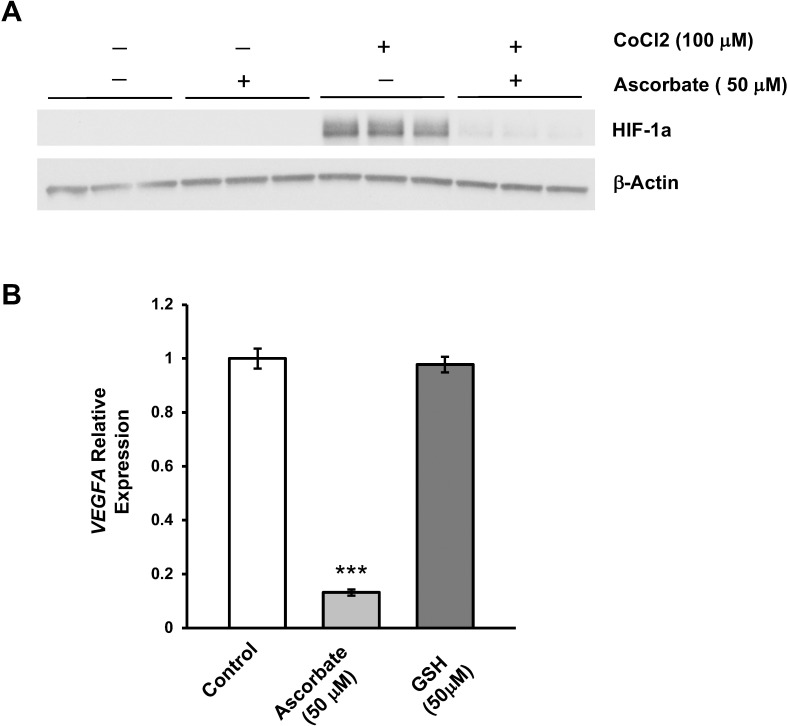
HIF-1α and antioxidant effects are not responsible for the suppression of VEGF by ascorbate in culture conditions. (A) Western blot shows that there was no detectable HIF-1α in cultured ARPE-19 unless induced by CoCl_2_ (100 μM) to mimic hypoxic conditions. After treatment with ascorbate (50 μM), the induced HIF-1α almost disappeared, but HIF-1α was not present in the experimental conditions of all other experiments. (B) qRT-PCR shows the suppression of VEGFA transcription by ascorbate, while a stronger reducing agent, glutathione (50 μM), exerted no obvious change in VEGFA transcripts in ARPE-19 cells (***P < 0.001).

HIF-1α is an important transcription factor that upregulates the expression of VEGF under hypoxic conditions.^31^ Degradation of HIF-1α requires ascorbate, which is also a cofactor for HIF-1α hydroxylases, enzymes that initiate the degradation of this transcription factor.^32^ Hence, the suppression of *VEGFA* by ascorbate could be mediated through an accelerated degradation of HIF-1α. To test this hypothesis, we measured HIF-1α levels in cultured ARPE-19 cells. HIF-1α was undetectable by Western blot in cultured ARPE-19 cells with or without ascorbate (Fig. 5A). This result was expected, since indoor air contains nearly 21% oxygen and the cell incubator environment contains 5% CO_2_. Addition of CoCl_2_, a compound mimicking a hypoxic environment, induced a significant increase in HIF-1α protein, which could be inhibited with ascorbate cotreatment (Fig. 5A).^27^ These results indicate that it is unlikely that the inhibition of VEGF by ascorbate is mediated by HIF-1α degradation since HIF-1α is not detectable in culture without hypoxia induction, yet ascorbate still reduces VEGF.

Alternatively, it is possible that ascorbate, a reducing agent, suppresses VEGF expression by attenuation of oxidative stress and associated ROS, which can upregulate VEGF in RPE cells.^33^ To rule out this possibility, ARPE-19 cells were treated for 7 days with either ascorbate or glutathione, a potent antioxidant. No inhibition of VEGF expression was seen in cells treated with glutathione (Fig. 5B). In contrast, VEGF expression was reduced to ∼11% of the control level in the presence of ascorbate. These findings rule out the possibilities that suppression of VEGF expression by ascorbate is mediated through either the HIF-1α pathway or attenuation of ROS, leading us to conclude that the decrease in VEGF is likely through the change in 5hmC levels in the gene body (Fig. 3).

### Ascorbate Reduces VEGF Expression in Primary Human Fetal RPE Cells

Although immortalized RPE cells (ARPE-19 and RPE-J) are convenient to grow for many passages and maintain a uniform cell homogeneity, they are not entirely representative of native RPE.^16^ In contrast, primary hfRPE are more difficult to grow and have higher cellular heterogeneity, but are an accurate representative of native RPE. They exhibit high levels of RPE markers, metabolize all-trans retinal, express high levels of pigmentation, polarize in culture, and develop high transepithelial resistance (TER).^16^

**Figure 6:**
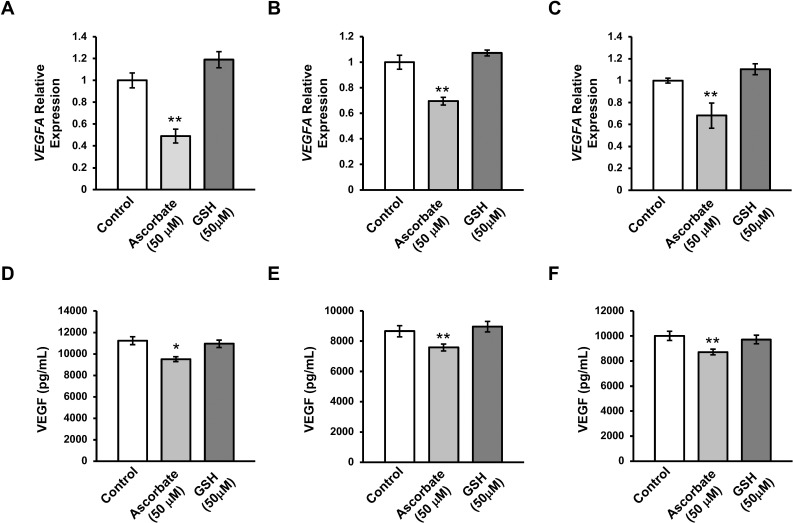
Ascorbate reduces the expression of VEGFA in primary hfRPE cells. qRT-PCR shows a reduction of VEGFA transcripts after treatment with ascorbate (50 μM), but not after treatment with glutathione (50 μM) in donors 1 through 3 (A–C). ELISA testing shows a reduction in the amount of secreted VEGF in the cell culture conditioned medium after treatment with ascorbate (50 μM), but not after treatment with glutathione (50 μM) in donors 1 through 3 (D–F) (*P < 0.05, **P < 0.01).

To test the effect of ascorbate in primary culture, RPE cells from three separate donors were obtained from the National Eye Institute (NEI). The initial culture media for hfRPE contains 250 μM L-ascorbic acid in its formulation. Following the first passage, cells were cultured with MEM media with the same formulation as the traditional MEM media used, except without the ascorbic acid. After 6 weeks of treatment with ascorbate-deficient media, cells were treated either with (50 μM) or without ascorbate for 7 days. By qRT-PCR, it was confirmed that the transcription of VEGF decreased after ascorbate treatment to ∼50% to 70% of the pretreatment levels in cells from all three donors (Figs. 6A–C). Additionally, ELISA on the conditioned culture medium revealed that levels of secreted VEGF from the cells of all three donors after ascorbate treatment were significantly reduced from the levels when ascorbate was absent. These experiments indicate that, similar to immortalized RPE cell lines, primary cells also downregulate VEGF in response to ascorbate treatment.

### Ascorbate Reduces VEGF in the Vitreous Humor of Mouse Eyes

To examine the effects of ascorbate on VEGF expression in vivo, we used *Gulo*^−/−^ mice. Unlike humans and other primates that cannot synthesize ascorbate due to loss-of-function mutations in the L-gulonolactone oxidase (*Gulo*) gene, rodents have fully functional L-gulonolactone oxidase, the enzyme that catalyzes the final step of ascorbate biosynthesis, and thus they can synthesize ascorbate de novo in the liver. Genetic ablation of the *Gulo* gene in rodents creates mice that, like humans, require vitamin C through their diet.^28^ The long-term survival of *Gulo*^−/−^ mice relies on ascorbate supplements. When supplemented with 330 mg/L ascorbate in the drinking water, the plasma ascorbate concentration of *Gulo*^−/−^ mice is maintained at ∼50 μM, similar to the level in wild-type mice.^28^

**Figure 7:**
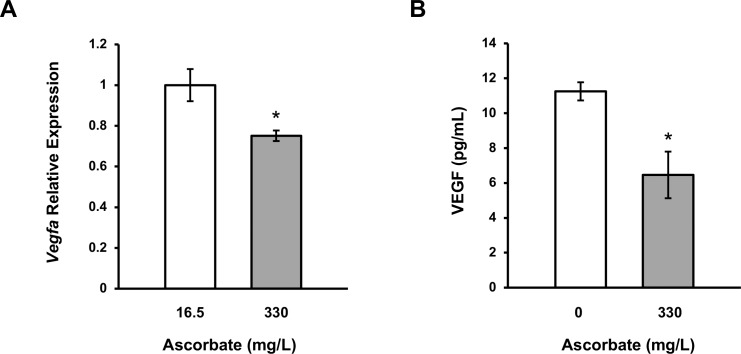
Decrease in the levels of VEGF in the RPE/choroid and vitreous humor of ascorbate-deficient mice supplemented with additional ascorbate. (A) qRT-PCR shows a reduction in Vegfa transcripts in the RPE/choroid layers of Gulo^−/−^ mice after being given ascorbate (330 mg/L) in the drinking water compared to mice kept with low ascorbate (16.5 mg/L) for 3 months. (B) The level of secreted VEGF protein, measured by ELISA, was decreased in the vitreous humor of Gulo^−/−^ mice given ascorbate (330 mg/L) in the drinking water compared to mice given no ascorbate for 4 months (*P < 0.05).

*Gulo*^−/−^ mice (1 month old) maintained with 330 mg/L ascorbate in their drinking water were divided into two random groups. The ascorbate supplementation was reduced to 16.5 mg/L for one group, but maintained at 330 mg/L for the other group. Three months later, mice were killed and the eyes were dissected. RNA was extracted from the RPE/choroid layers and the levels of *Vegfa* transcripts were measured by qRT-PCR. The mice with high ascorbate supplementation showed a significantly reduced level of *Vegfa* transcripts (0.75-fold) in the RPE/choroid layers compared to those mice given low levels of vitamin C (*P* = 0.031, Fig. 7A).

In a second experiment, *Gulo*^−/−^ mice (3 months old) maintained with 330 mg/L ascorbate in their drinking water were divided into two random groups. Ascorbate supplementation was removed from one group while the ascorbate supplementation was continued for the other group of mice. Four months later, mice were killed and VEGF protein level was measured in the vitreous humor by ELISA. The *Gulo*^−/−^ mice with ascorbate supplementation showed a significant decrease of VEGF (0.57-fold) accumulated in their vitreous humor in comparison to those without ascorbate supplementation (*P* = 0.029, Fig. 7B). These in vivo data indicate that ascorbate inhibits VEGF expression in the eye, suggesting that our in vitro studies do mirror in vivo conditions. Further studies with large sample sizes will determine if ascorbate-induced changes in VEGF levels lead to alterations in retinal vasculature.

## Discussion

Results of this study showed the ability of ascorbate to effectively suppress VEGF transcription and protein secretion in RPE cells. The suppression of VEGF by ascorbate was initially discovered in human ARPE-19 cells, then verified in rat RPE-J cells and in primary hfRPE cells, suggesting that ascorbate inhibits VEGF generally in RPE cells. Additional experimentation showed that glutathione, a potent reducing reagent with antioxidant properties comparable to those of ascorbate, was not sufficient to reduce VEGF expression. This reduction of VEGF occurs in the absence of HIF-1α expression, suggesting that it is independent of the HIF-1α pathway. Our results indicate that a 5hmC increase in the gene body, specifically intron 2, correlates with the reduced expression of VEGF by ascorbate. Generally, suppression of a gene is more frequently associated with an increase of 5hmC in the promoter, but previous reports have shown that changes in the gene body of a gene can also be correlated with a decrease in gene transcription.^34^ It is noteworthy that a single nucleotide polymorphism (SNP) rs833069 within intron 2 is associated with the risk of AMD.^35^ This SNP is located in close proximity to the differentially hydroxymethylated region, raising a possibility that this variant may affect VEGF expression via DNA hydroxymethylation. Furthermore, ascorbate supplementation was found to decrease VEGF levels in the RPE/choroid and vitreous humor of *Gulo*^−/−^ mice, suggesting that as in vitro, RPE cells in vivo have reduced secretion of VEGF with ascorbate. Thus the present work suggests a major contributory role of ascorbate in regulating VEGF expression in the retina.

It is generally agreed that oxidative stress, which is associated with elevated levels of ROS, contributes to the pathogenesis of AMD.^11^ One consequence of the high level of ROS is depletion of ascorbate in the retina by oxidizing ascorbate to DHA, which has no function for the TET enzymes. The lack of a cofactor for TET enzymes in turn could decrease 5hmC levels in the gene body of *VEGF*, leading to an increase in the transcription of *VEGF*. As a consequence, VEGF secretion increases, which may contribute to the pathological angiogenesis in the retina, including wet AMD.

Diabetes has also been associated with both elevated ROS in the retina and increased levels of VEGF.^36^ Anti-VEGF therapies have been shown to be effective for a subset of patients with diabetic retinopathy and diabetic macular edema.^37–39^ Additionally, hyperglycemia has been shown to inhibit uptake of ascorbate by the retina, and studies have shown that the ascorbate levels in the vitreous humor of patients with diabetic retinopathy are only approximately 30% as high as in nondiabetic patients.^40,41^ These data indicate that ascorbate plays a role in the suppression of VEGF in diabetic eye complications.

Identification of the critical role VEGF plays in retinal pathological angiogenesis has led to the development of anti-VEGF therapies that have significantly changed the treatment of wet AMD and diabetic vision complications. Current anti-VEGF therapies, using either antibodies (such as Lucentis, Avastin) or soluble decoy receptors (such as Eylea), target the expressed VEGF protein to make it less available, but do not target VEGF expression at transcriptional, translational, or secretory levels. Our results show that ascorbate inhibits VEGF at the transcriptional level, which not only gives new insight into pathological retinal angiogenesis disorders, but also could be included in new prevention and treatment strategies for those diseases. Advanced age is a risk factor in developing AMD and diabetes, but additionally, tissue ascorbate levels generally decline with age.^42^ For example, the ascorbate level in leukocytes is approximately 50% as high in individuals age 85 and older as in those at age 60.^43^ These data suggest that low levels of ascorbate in tissue are a comorbidity of diseases like AMD and diabetes, and that supplemental levels will be needed to maintain VEGF expression at healthy levels.

Overall, ascorbate, a well-tolerated micronutrient, could be implicated in the prevention or treatment of VEGF-mediated retinal diseases such as AMD and diabetic eye disease.

## Supplementary Material

Supplement 1

Supplement 2

Supplement 3

Supplement 4
