# Supplementary material for: Ascorbate Suppresses VEGF Expression in Retinal Pigment Epithelial Cells
Source: Invest Ophthalmol Vis Sci. 2018 Jul;59(8):3608–18. doi: 10.1167/iovs.18-24101 (PMC6049987; doi:10.1167/iovs.18-24101)
Supplement: Supplement 3 [file iovs-59-08-22_s03.pdf]

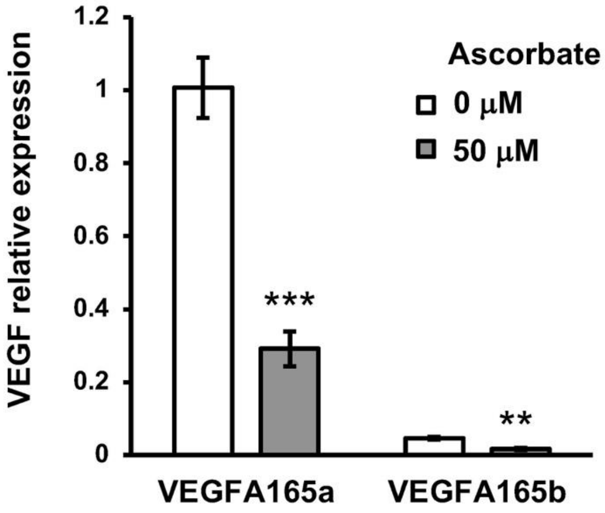

Supplemental Figure 1. Both isoforms of *VEGFA* were reduced after ascorbate treatment.

The transcript level of pro-angiogenic *VEGFA*-165a was much higher than that of anti-angiogenic *VEGFA*-165b, both of which were suppressed by ascorbate (50 μM) treatment. (** $P < 0.01$, *** $P < 0.001$)
