# Supplementary material for: Ascorbate Suppresses VEGF Expression in Retinal Pigment Epithelial Cells
Source: Invest Ophthalmol Vis Sci. 2018 Jul;59(8):3608–18. doi: 10.1167/iovs.18-24101 (PMC6049987; doi:10.1167/iovs.18-24101)
Supplement: Supplement 4 [file iovs-59-08-22_s04.pdf]

Supplemental Table 1. Primers used for quantitative RT-PCR.

Gene	Species	Forward (5′→3′)	Reverse (5′→3′)
VEGFA	Human	GCCTTGCCTTGCTGCTCTAC	TGATTCTGCCCTCCTCCTTCTG
VEGFA 165-a	Human	GAGCAAGACAAGAAAATCCC	CCTCGGCTTGTCACATCTG
VEGFA 165-b	Human	GAGCAAGACAAGAAAATCCC	GTGAGAGATCTGCAAGTACG
ACTB	Human	TCCCTGGAGAAGAGCTACG	GTAGTTTCGTGGATGCCACA
Vegfa	Rat	GAGCAACGTCACTATGCAGATCATGCG	CTTTGGTCTGCATTCACATCTGCTATGCTG
Actb	Rat	GCCACCAGTTCGCCATGGATGACG	CACACCCRGGRGCCRAGGGCGG
Vegfa	Mouse	GCCAGCACATAGGAGAGATGAGCTTCC	CTTTGGTCTGCATTCACATCTGCTGTGC
Actb	Mouse	GTCGAGTCGCGTCCACC	GTCATCCATGGCGAACTGGT
